# Depletion of polycistronic transcripts using short interfering RNAs: cDNA synthesis method affects levels of non-targeted genes determined by quantitative PCR

**DOI:** 10.1186/1743-422X-10-159

**Published:** 2013-05-21

**Authors:** Jennifer E Hanning, Ian J Groves, Mark R Pett, Nicholas Coleman

**Affiliations:** 1 Department of Pathology, University of Cambridge, Tennis Court Road, Cambridge CB2 1QP, UK

**Keywords:** Quantitative PCR, Short interfering RNA, cDNA synthesis

## Abstract

**Background:**

Short interfering RNAs (siRNAs) are often used to deplete viral polycistronic transcripts, such as those encoded by human papillomavirus (HPV). There are conflicting data in the literature concerning how siRNAs targeting one HPV gene can affect levels of other genes in the polycistronic transcripts. We hypothesised that the conflict might be partly explained by the method of cDNA synthesis used prior to transcript quantification.

**Findings:**

We treated HPV16-positive cervical keratinocytes with siRNAs targeting the HPV16 E7 gene and used quantitative PCR to compare transcript levels of E7 with those of E6 and E2, viral genes located upstream and downstream of the target site respectively. We compared our findings from cDNA generated using oligo-dT primers alone with those from cDNA generated using a combination of random hexamer and oligo-dT primers. Our data show that when polycistronic transcripts are targeted by siRNAs, there is a period when untranslatable cleaved mRNA upstream of the siRNA binding site remains detectable by PCR, if cDNA is generated using random hexamer primers. Such false indications of mRNA abundance are avoided using oligo-dT primers. The period corresponds to the time taken for siRNA activity and degradation of the cleaved transcripts. Genes downstream of the siRNA binding site are detectable during this interval, regardless of how the cDNA is generated.

**Conclusions:**

These data emphasise the importance of the cDNA synthesis method used when measuring transcript abundance following siRNA depletion of polycistronic transcripts. They provide a partial explanation for erroneous reports suggesting that siRNAs targeting HPV E7 can have gene-specific effects.

## Findings

Gene depletion using siRNAs is an important tool in biological investigations and clinical therapeutics. It is often applied for depleting polycistronic transcripts, particularly those derived from viruses [1]. One example is high-risk human papillomavirus (HR-HPV), the necessary cause of cervical carcinoma [2]. Numerous siRNAs have been designed to target the major HR-HPV oncogenes E6 and E7. These genes are expressed in multiple polycistronic transcripts, in which E6 is upstream of E7 [3]. In cells where HR-HPV is integrated into host DNA the transcripts often contain E6 and E7 only, whereas in cells where the virus remains extra-chromosomal (episomal) there are additional downstream genes, including E2. There are conflicting reports regarding the effect of particular siRNAs on expression of other genes in the polycistronic transcripts, as assessed using quantitative reverse-transcription PCR (qRT-PCR). While some studies show that siRNAs targeting E7 also deplete E6 (and vice versa) [4,5], there is also widely-cited evidence that siRNAs can specifically deplete E7 without affecting E6 [6].

We hypothesised that the different results reported may be partly explained by the method used to generate cDNA prior to PCR quantification of individual HR-HPV oncogenes. Binding of siRNAs can result in cleaved transcripts that are subsequently degraded, at a rate that varies between systems [7]. While mRNA upstream of the cleavage site would not be translated, as it has no polyA tail, the genes encoded would still be detectable by PCR if cDNA was generated using random hexamer primers. In contrast, mRNA upstream of the cleavage site would not be detectable if cDNA was generated using oligo-dT primers, which would only generate cDNA from downstream of the cleavage site.

**Figure 1:**
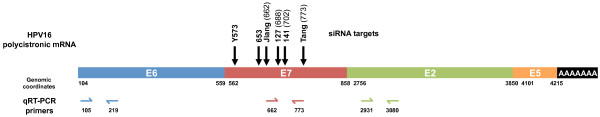
**Location of siRNA and PCR primer binding sites.** A representative HPV16 polycistronic transcript, showing the binding sites of the siRNAs and PCR primers used in the present study. Full details of the range of HPV16 polycistronic transcripts are provided elsewhere [3].

We initially tested this hypothesis by treating the HPV16-positive cervical squamous cell carcinoma (SCC) cell line CaSki [8] with siRNAs targeting HPV16 E7 and measuring levels of E7 and the upstream gene E6. We used six different siRNAs that mapped along the E7 open reading frame (Table 1 and Figure 1) [9]. Cells were transfected according to manufacturer’s instructions using Lipofectamine RNAiMAX (Invitrogen, Paisley, UK) and 9nM siRNA. To control for non-specific siRNA effects, a pool of non-targeting control siRNAs (ON-TARGET plus non-targeting, Thermo Scientific) was used, also at 9nM. RNA was extracted using TRIzol (Life Technologies, Grand Island, NY, USA) according to manufacturer’s instructions. Twenty-four hours after each treatment, cDNA was generated using two different methods: a combination of random hexamer and oligo-dT primers (QuantiTect reverse transcription kit, Qiagen, Crawley, UK), and oligo-dT primers alone (Qiagen, UK). Levels of HPV16 E7 and E6 were then measured by qRT-PCR as described [9], using the primers listed in Table 2. Primer binding sites are illustrated in Figure 1.

**Table 1:** Sequences of siRNAs targeting HPV16 E7

**siRNA source**	**Name**	**siRNA sense (passenger) strand sequence**
Tang [4]	Tang	GCACACACGUAGACAUUCG
Dharmacon	127	GGACAAGCAGAACCGGACA
Dharmacon	141	GGACAGAGCCCAUUACAAU
Dharmacon	653	GCUCAGAGGAGGAGGAUGA
Yamato [5]	Y573	CACCUACAUUGCAUGAAUA
Jiang and Milner [6]	Jiang	AGGAGGAUGAAAUAGAUGG

**Table 2:** **Primers used for qRT**-**PCR**

**Target**	**Forward primer (5’ to 3’)**	**Reverse primer (5’ to 3’)**
HPV16 E7	AGGAGGATGAAATAGATGGTCC	CTTTGTACGCACAACCGAAGC
(nt 662–773)		
HPV16 E6	AGCGACCCAGAAAGTTACCA	GCATAAATCCCGAAAAGCAA
(nt 105–219)		
HPV16 E2	GGAGACTCTTTGCCAACGTTTA	CACATTCTAGGCGCATGTGT
(nt 2931–3080)		
GAPDH	TGCACCACCAACTGCTTAGC	GGCATGGACTGTGGTCATGAG
TBP	Hs_TBP_1_SG (Qiagen)	
ACTB	Hs_ACTB_2_SG (Qiagen)	

**Figure 2:**
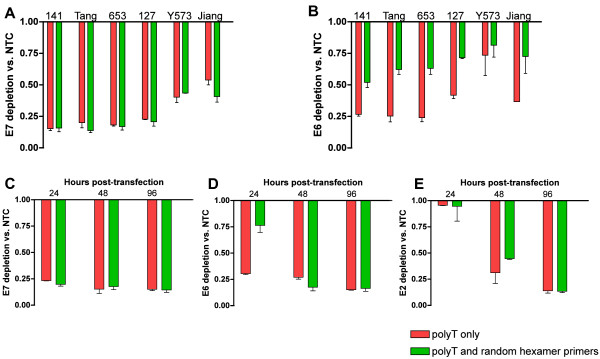
**RT-PCR quantification of HPV16 mRNA depletion in E7-siRNA treated cells.** The top row shows levels of **A**) E7 and **B**) E6 24 hours after treatment of CaSki with E7-targeting siRNAs. The bottom row shows levels of **C**) E7, **D**) E6 and **E**) E2 at 24, 48 and 96 hours after treatment of W12Ser4p86 with E7-141 siRNAs. Each pair of bars shows levels detected in the same cells using cDNA generated by oligo-dT primers only (red) or oligo-dT and random hexamer primers (green). Error bars show the standard deviations, based on three independent experiments. NTC = non-targeting control.

Across the siRNAs, the mean level of E7 depletion measured at 24 hours was 74.7% (range 59.2-84.2%) for cDNA made with random hexamer and oligo-dT primers and 71.6% (46.1-84.8%) for cDNA made with oligo-dT primers alone (Figure 2A). For cDNA made using oligo-dT primers only, levels of E6 mirrored those of E7 (Figure 2B). In contrast, for all six siRNAs, when using cDNA made with combined random hexamer and oligo-dT primers, E6 reductions were significantly lower than E7 (Figure 2B), with a mean reduction of 41.9% (range 31.8-50.8%) (*P*-*value* = 0.00011, t-test). Such differences between E6 and E7 levels were greatest for the siRNAs that produced the greatest depletion of E7, although the trend was maintained for all siRNAs.

The most potent siRNA, E7-141, was subsequently optimised for transfection in a different HPV16-containing cervical squamous cell line, W12 series-4 passage-86 (W12Ser4p86) [10]. These cells reform an SCC in organotypic tissue culture and stably retain approximately 500 episomes of HPV16 per cell. They therefore express polycistronic transcripts in which E6 is upstream of E7, with other early genes including E2 downstream [10]. We predicted that levels of E6 depletion determined following cDNA synthesis using oligo-dT primers would be greater than those determined following cDNA synthesis using random hexamer primers but that depletion of E2 would be similar for both cDNA synthesis methods. Furthermore, measuring the disparities in viral gene levels over time would allow us to detect the interval over which siRNA activity and degradation of cleaved transcripts occurred. Accordingly, we measured of E7, E6 and E2 at 24, 48 and 96 hours following E7-141 treatment of W12Ser4p86.

As with CaSki, E7-141 treatment produced mean E7 depletion levels of 82.4%, which did not vary significantly over the time-period studied and did not differ according to cDNA synthesis method (Figure 2C). At 24 hours following treatment the levels of E6 depletion detected in cDNA generated using oligo-dT alone were similar to those determined for E7 (70.0% for E6, 76.6% for E7: Figure 2C and D), whereas the levels of E6 depletion determined in cDNA generated by random hexamers and oligo-dT were significantly less (23.6%, *P*-*value* = 0.01359, t-test) (Figure 2D). Levels of E2 depletion at 24 hours were very low (mean = 4.9%), regardless of the cDNA synthesis method (Figure 2E). In contrast, at 48 and 96 hours, levels of E6 and E2 depletion determined by PCR were similar to those of E7, regardless of the cDNA synthesis method (Figure 2C-E).

**Figure 3:**
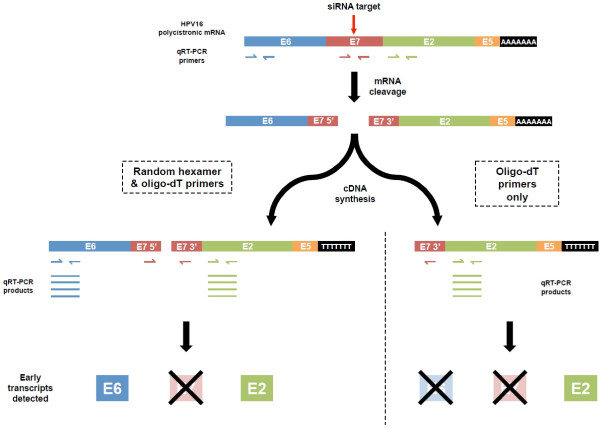
**Model for the effects of cDNA synthesis method on PCR quantification of polycistronic transcript genes following siRNA treatment.** The Figure shows the representative early HPV16 polycistronic transcript from Figure 1. Following treatment with E7-targeting siRNA, the transcript is cleaved around the siRNA binding site (second row). If PCR transcripts are quantified using cDNA generated with random hexamer and oligo-dT primers (left side), genes upstream of the cleavage site (in this schematic, E6) will be detected even though they are not translatable, until siRNA activity and degradation of the cleaved fragments is complete. In contrast, using cDNA generated with oligo-dT primers alone (right side) will not lead to false detection of such upstream genes. Both methods of cDNA synthesis will lead to misleading PCR detection of genes downstream of the cleavage site (in this schematic, E2), until completion of siRNA activity and fragment degradation. For both cDNA synthesis methods the cleaved target gene (in this schematic, E7) will not be detected by PCR.

Our data are consistent with the model shown in Figure 3. Treatment with E7-targetting siRNAs leads to cleavage of the HPV16 polycistronic transcripts around the site of binding, preventing detection of the target gene E7 regardless of the mode of cDNA synthesis. However, as siRNA activity and degradation of the cleaved transcripts progress, there is a window (between 24 and 48 hours in our W12 experiment) where misleading information may be obtained. For genes in the transcript that are upstream of the siRNA binding site (E6 in our experiments), PCR quantification using cDNA generated by random hexamers will detect untranslatable transcripts and so give an inappropriately high indication of gene abundance. A more appropriate measure of the abundance of such upstream genes would be provided by cDNA generated using oligo-dT primers alone. For genes in the polycistronic transcript that are downstream of the siRNA binding site (such as E2), cDNA generated using either random hexamers or oligo-dT primers would give a falsely high indication of gene abundance, until siRNA activity and degradation of the cleaved transcript is complete.

Our data may provide at least a partial explanation for conflicting observations in the literature concerning the specificity of siRNAs targeting HPV16 E7. The previous study that showed evidence of E7-specific effects [6] is likely to have quantified E6 in cleaved but undegraded mRNAs, as random decamers were included in the *Reverse*-*iT* reverse transcription kit used. It should be noted that differences in E6 versus E7 levels in the previous study persisted to 48 hours in CaSki and SiHa cells, in contrast to our present findings in W12. There was also continued suppression of p53 protein until 72 hours in cells treated with E7 siRNAs, although the presumption that this represented the sustained presence of E6 protein was not tested directly [6]. These caveats aside, our data suggest that when siRNAs are used to target any polycistronic mRNA transcript (i.e. not just those derived from HPV), careful consideration should be given to the method of cDNA synthesis and the relative binding sites of the PCR primers and siRNAs used.

## Competing interests

The authors declare that they have no competing interests.

## Author contributions

JEH carried out all molecular biology work, contributed to design of the study, was involved in all data analysis and interpretation and drafted the manuscript. IJG was involved in data analysis and manuscript preparation. MRP and NC conceived the study and participated in its design and interpretation of data analysis. NC co-wrote the manuscript. All authors read and approved the manuscript.
